# Synthesis and Characterization of Ni Nanoparticles via the Microemulsion Technique and Its Applications for Energy Storage Devices

**DOI:** 10.3390/ma16010325

**Published:** 2022-12-29

**Authors:** Zia Ur Rehman, Mohsan Nawaz, Hameed Ullah, Imad Uddin, Salma Shad, Elsyed Eldin, Razan A. Alshgari, Aboud Ahmed Awadh Bahajjaj, Waqas Ul Arifeen, Muhammad Sufyan Javed

**Affiliations:** 1 Department of Chemistry, The University of Haripur, Haripur 22620, Pakistan; 2 Department of Chemistry, Hazara University Mansehra, Mansehra 21120, Pakistan; 3 Department of Chemistry, Islamia College University Peshawar, Peshawar 25120, Pakistan; 4 Faculty of Engineering and Technology, Future University in Egypt, New Cairo 11835, Egypt; 5 Chemistry Department, College of Science, King Saud University, Riyadh 11451, Saudi Arabia; 6 School of Mechanical Engineering, Yeungnam University, Gyeongsangbuk-do, Gyeongsan-si 38541, Republic of Korea; 7 School of Physical Science and Technology, Lanzhou University, Lanzhou 730000, China

**Keywords:** supercapacitor, nickel nanoparticles, microemulsion, positive electrode

## Abstract

Herein, a unique synthetic approach called microemulsion is used to create nickel nanoparticles (Ni-NPs). SEM, TEM, EDX, and XRD techniques were employed for the investigation of morphology and structures of the synthesized material. Electrons from electroactive components are transferred to external circuits by Ni-NPs’ superior electrical conductivity and interconnected nanostructures, which also provide a large number of channels for ion diffusion and additional active sites. The experimental findings showed that as a positive electrode for supercapacitors (SC), Ni-NPs had an outstanding ability to store charge, with a dominant capacitive charge storage of 72.4% when measured at 10 mV/s. Furthermore, at 1 A/g, Ni-NP electrodes exhibit a maximum capacitance of 730 F/g. Further, the Ni-NP electrode retains 92.4% of its capacitance even for 5000 cycles, highlighting possible applications for it in the developing field of renewable energy. The current study provides a new method for producing high-rate next-generation electrodes for supercapacitors.

## 1. Introduction

The problems of environmental deterioration and carbon emissions are relevant to human society. To address these issues, much emphasis has been paid to renewable energy sources, including tidal, solar, and wind. However, because these energy sources are intermittent, efficient and durable energy storage systems are in greater demand [1,2,3,4]. Novel high-efficiency energy storage technologies have been developed in response to the increasing need for real-world applications demanding high power/energy densities, including portable electronics and electric cars [5,6]. Two extensively employed electrochemical energy systems are supercapacitors (SCs) and rechargeable batteries. SCs are stable, have a long lifespan and quick charging–discharging, and require less maintenance cost [7,8,9]. Electrode materials are obviously significant in the fabrication of SCs regardless of the charge storage method. Recently, few strategies have been proven effective and are frequently used to create high-rate active materials with excellent porosity and a wide range of redox states for enhanced conductivity and high performance. It provides electrode materials with sufficient kinetics for charge transfer, allowing active materials to employ their storage capacity, thus increasing power density and energy density [10].

Recent studies have identified that transition metal-oxide-based materials as favorable active materials for SCs [11,12,13,14]. Among them, TiO_2_ [15], MnO_2_ [16], and SnO_2_ [17] have been employed for SC applications because of their excellent specific capacitance and number of oxidation states for effective redox processes. With the advancement of nanotechnology, more efforts have been made for large-scale nanoparticle production due to their potential applications in many areas [18,19,20,21]. NPs’ size, shape, and structure will greatly influence their performance in different devices. The electrochemical performance of NP-based electrodes is significantly influenced by their morphological structure, which includes their size, shape, and structure [22,23]. NPs’ preparation with a low cost and desired quality via convenient methods is of great value. Ni is an important transition metal, and its NPs possess several applications [24]. Because of such applications, Ni-NPs have been a matter of great interest for scientists. Various methods have been adopted for Ni-NP synthesis, but few of them are used for large-scale preparation [25,26,27,28,29,30,31,32]. Pfeil et al. prepared Ni powder from nickel carbonyl decomposition and via electrochemical reduction [33]. The microemulsion method is most important and has many advantages over other methods because of its easy handling, inexpensiveness, and convenience [32,34,35,36,37,38,39]. There are only a few synthesis approaches for Ni-NPs that have been published on a large scale using microemulsion techniques. To control size and shape, improvements in Ni-NP synthesis processes are required to obtain for the desired applications. Ni-NPs are the most challenging metal NPs to synthesize because they oxidize readily. Numerous techniques have been used in organic mediums to create pure nickel nanocrystals and prevent the production of oxide or hydroxide [36,40,41,42,43]. 

This research outlined a novel synthetic method, microemulsion, for producing Ni-NPs. Ni-NP electrodes show a maximum capacitance of 730 F/g at 1 A/g and preserve 92.4% of their capacitance even after 5000 cycles, suggesting their potential use in the expanding field of renewable energy. 

## 2. Experimental Method

### 2.1. Preparation of Ni-NPs 

**Figure 1:**
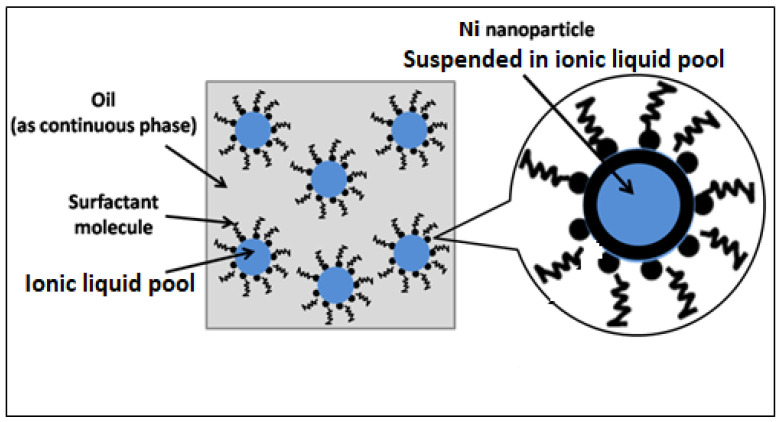
Microemulsion synthetic route of Ni-NP preparation.

To synthesize Ni-NPs, microemulsion techniques were used, and the NPs’ size varied as a function of microemulsion parameters. Sodium borohydride/hydrazine was used for the reduction of Nickel (II) in a droplet of microemulsion. Using 6.5 g (Dioctyl sodium sulfosuccinate (AOT or Aerosol-OT)), 13.5 g (n-heptane) and 4.5 g ionic liquid contain 1 M Ni (II) and 3 M sodium borohydride. The procedure for the reaction was adapted and scaled down, as stated elsewhere [44]. The reduction of Ni was completed when dark black color appeared. Finally, the product was separated using a centrifuge, after which it was completely cleaned with commercial ethanol dry at 60 °C. The composition of the microemulsion consisted of three elements: an ionic liquid dispersant, an oil continuous phase, and neutral, anionic, or cationic surfactant. Ionic liquid was employed to prevent the oxidation of the synthesized NPs. By varying the microemulsion parameters, the size and shape of NPs were controlled. Further, to avoid oxidation overall, the reaction was performed inside an argon-filled glovebox, and inside the glovebox, water vapor and oxygen level were kept below 1 ppm. Figure 1 depicts the microemulsion procedure used to create Ni-NPs. 

### 2.2. Physical Characterization

An X-ray diffraction (XRD) test was conducted with a Huber G670 diffractometer using Mo-Kα radiation (GmbH % Co. KG, Rimsting, Germany). The surface architecture and morphology were tested using an Oxford Instruments EDX INCA SYSTEM mounted on a Zeiss LEO 1530 scanning electron microscope (SEM), energy-dispersive X-ray spectroscopy (EDX), and a transmission electron microscope (TEM).

### 2.3. Electrode Fabrication and Electrochemical Characterization

For three-electrode configuration, the working electrode was composed of active materials, conductive carbon black, and polyvinylidene fluoride (PVDF) as a binder, in the mass ratio of 80:10:10 (wt. %), respectively. N-Methyl-2-Pyrrolidone (NMP) was employed as a solvent to make the uniform slurry, which was then pasted onto 1 × 1 cm^2^ carbon cloth (CC) and dried at 60 °C. About 1.3 mg/cm^2^ of the active material was loaded onto CC. In a 6 M potassium hydroxide (KOH) solution, platinum wire served as a counter electrode, while Ag/AgCl served as a reference electrode. The cyclic voltammogram (CV) and galvanostatic charge–discharge (GCD) tests were carried out on an electrochemical workstation (CHI 660E, China). The GCD tests were conducted at different current densities between 1 and 10 A/g, whereas the CV investigations were conducted in a potential window range of 0.0 to 0.6 V.

The capacitance *C_sp_* (*F/g*) and the Coulombic efficiency (*η*) of the electrode are determined as [45,46]:

(1) $$C_{sp}=\frac{I\Delta t}{m\times \Delta V}$$

(2) $$\eta \;\left(\%\right)=\frac{\Delta t_{d}}{\Delta t_{c}}\times 100$$

where Δ*t* (*s*) denotes the discharge time, *I* (*A*) denotes the current, Δ*V*(*V*) denotes the potential window, *m* (*g*) denotes the mass of electrode, and Δ*t_c_* and Δ*t_d_* are charging and discharging time.

The energy density (*E*, *Wh/kg*) and power density (*P*, *W/kg*) of electrode are determined as [47,48,49]:

(3) $$E=\frac{1}{2}C_{d}V^{2}\times \frac{1000}{3600}$$

(4) $$\;P=\frac{E}{\Delta t}$$

## 3. Results and Discussion

**Figure 2:**
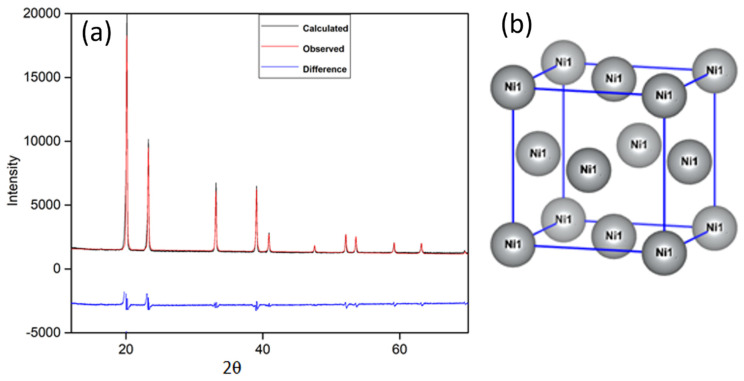
(**a**) XRD patterns of Ni-NPs; (**b**) crystal structure of cubic Ni-NPs.

To examine the crystal structure of the synthesized sample, X-ray diffraction (XRD) was performed. Figure 2a shows the XRD patterns of the fresh Ni-NPs, which revealed the face-centered cubic (FCC) crystal structure of Ni. Four characteristic peaks at 20.020°, 23.150°, 32.972°, and 38.860° were observed, which corresponds to (111), (200), (220), and (311) crystal planes of Ni (JCPDS, no. 03–1051), respectively. This demonstrates pure Ni-NPs and the intensity of these increases with increases in size of Ni-NPs. According to the Scherrer formula (Equation (5)), the principle diffraction peaks of the base of (111) were used to determine the crystallite size (*D* (μm)) of the as-prepared Ni [15].

(5) $$D=\frac{K\lambda }{\beta \cos\theta }$$

where *D* (μm) is the crystallite diameter, *λ* (nm) denotes the wavelength of X-rays, and *β* denotes the diffraction peak’s whole width at half maximum. The crystallite size of NPs calculated by the Scherrer formula is 14 nm. Figure 2b shows the crystal structure of the FCC of Ni-NPs. 

**Figure 3:**
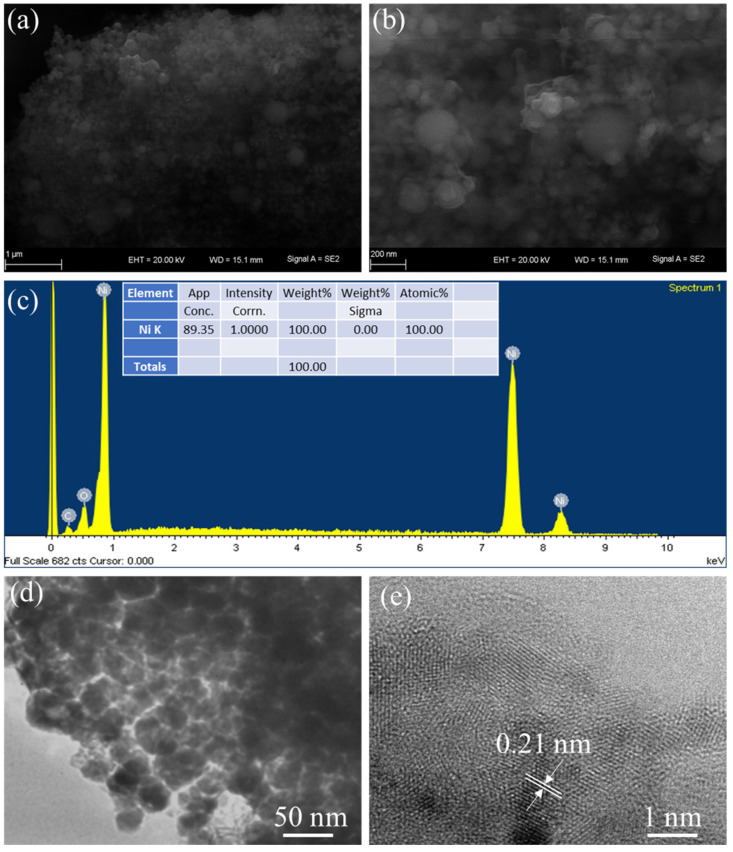
(**a**,**b**) Low- and high-resolution SEM images of Ni-NPs; (**c**) EDX elemental mapping; (**d**,**e**) low- and high-resolution TEM images of Ni-NPs.

For the study of morphological characterizations of Ni-NPs, SEM was performed. Figure 3a,b show SEM images at low and high resolution, which exhibit that Ni-NPs have a spherical shape and particles were uniformly distributed. The mean size of Ni-NPs was determined to be 9–15 nm. The pore-free crystallite on the surface indicates the highly dense agglomeration. The images show the agglomeration of Ni-NPs. The agglomeration and growing assembly size of the NPs are due to high surface energy. Further, agglomeration was observed with a cluster of NPs. The high surface area of particles enhances the energy storage properties. EDX analysis confirmed that the elementary component was Ni-NPs, as shown in Figure 3c. 

For the further investigation of the morphological characterizations of prepared sample, TEM was used. Figure 3d shows a TEM image of Ni-NPs, which exhibit primarily spherical Ni-NPs with a restricted size distribution. In fact, it was observed that the small crystallites further arrange themselves in a cluster of hierarchal structure. Further, a TEM micrograph observed that the particle size was spherical, while some agglomerated and elongated particles were also present. The particle size of the prepared sample was 9–15 nm, which was in correlation with XRD values. The high-resolution TEM (HRTEM) image of Ni-NPs clearly exhibits fringes with 0.21 nm spacing, which corresponds to the plane of (103) of Ni-NPs.

**Figure 4:**
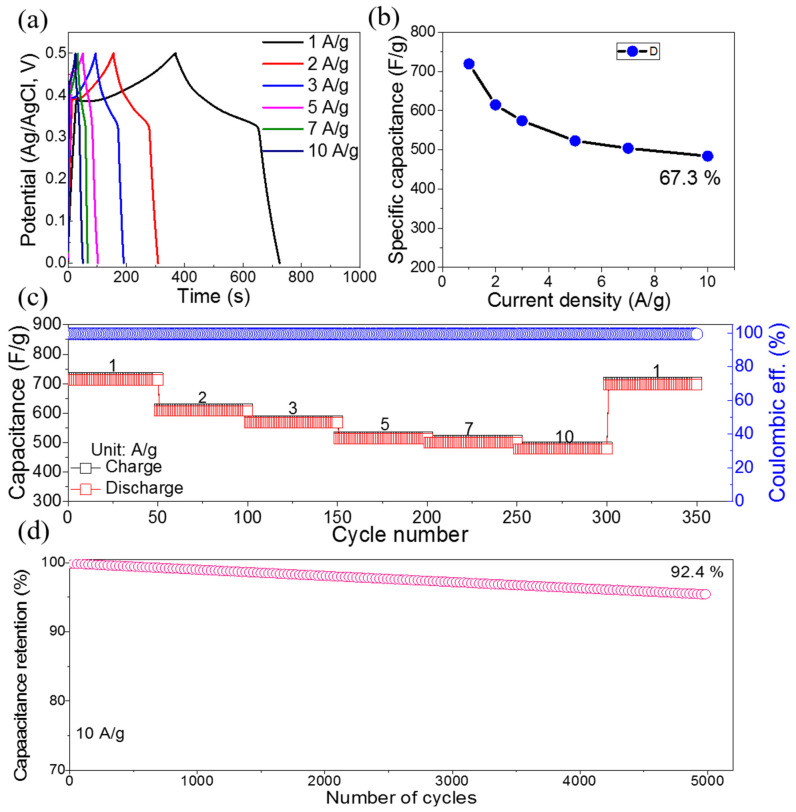
(**a**) GCD profiles of Ni-NPs; (**b**) specific capacitance vs. current density; (**c**) rate performance (left side) and Coulombic efficiency (right side); (**d**) cycling stability.

The Ni-NPs were investigated as positive electrodes for SC, and electrochemical tests were carried out in a three-electrode system. Figure 4a shows the GCD profiles of the Ni-NPs at various current densities (1 to 10 A/g) and potential window ranges of 0.0–0.5 V. The GCD profiles clearly exhibit outstanding symmetry, showing great Coulombic efficiency and extraordinary electrochemical activity. In addition, two voltage plateaus were detected in the GCD profiles because of the redox reaction occurring in charging and discharging. Further, the Ni-NPs show a capacitance of 730 F/g at 1 A/g (as found by Equation (1)), as shown in Figure 4b. Because of insufficient electrode material participating in the redox process at increasing current densities, electrode capacitance continuously decreases as current density rises. However, at 10 A/g, capacitance retained as much as 67.3% of its capacitance. Furthermore, the Ni-NP electrode maintained its exceptional rate performance even for 1 to 10 A/g (Figure 4c). It is impressive that the capacitance recovered nearly ~99% for 10 to 1 A/g for 350 cycles. Additionally, the Coulombic efficiency of electrode approached ~100% (Figure 4c). The cyclic stability 10 A/g shows that the Ni-NP electrode exhibited a retention of 92.4% for 5000 cycles, as illustrated in Figure 4d. Thus, the Ni-NP electrode has an exceptional cyclic performance with ~100% Coulombic efficiency. It verifies the Ni-NP electrode’s exceptional capability to store energy across numerous charge–discharge cycles. Additionally, Table 1 summarizes the performance of the Ni-NPs in comparison to other nanostructured electrodes. The comparison shows that the Ni-NP electrode material’s electrochemical performance is better than those of already researched similar electrodes.

**Figure 5:**
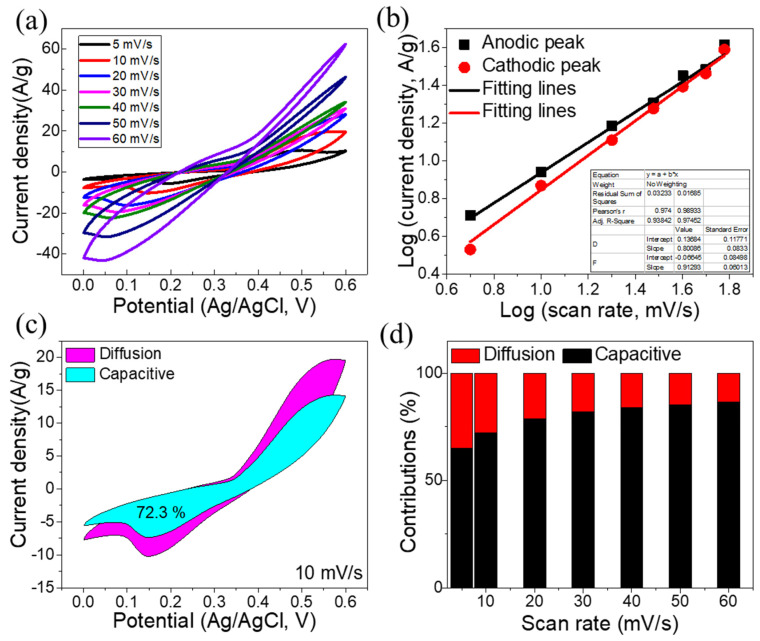
(**a**) CVs of Ni-NPs; (**b**) *b*-value’s calculations; (**c**) capacitive/diffusion-controlled charge store at 10 mV/s, (**d**) capacitive/diffusion-controlled charge store at various scan rates (5–60 mV/s).

Figure 5a reveals the results of CV measurements performed on the Ni-NPs at various scan rates (5–60 mV/s). The appearance of two redox peaks in each of these profiles was recorded, which demonstrates the exceptional capacitive performance of the Ni-NP electrode. 

Furthermore, the method to store charge for Ni-NPs was examined by the power law [50,51].

*i*(*V*) = *a.v^b^* (6)

log (*i*) = *b* log (*v*) + log (*a*) (7)

When the scan rate is denoted by *v*, the peak current density is denoted by *i* and arbitrary constants are denoted by a, b. Generally, the diffusion-controlled method will prevail for a *b*-value of 0.5, and the capacitive-controlled method will prevail if it reaches 1.0 [51]. These *b*-values are determined and the anodic *b*-value for Ni-NPs is 0.80 and cathodic *b*-value is 91, as illustrated in Figure 5b. This implies that the overall charge storage was influenced by both capacitive- and diffusion-controlled methods. Moreover, Figure 5c shows that at 10 mV/s, the Ni-NP electrode stores 72.3% of its charge via a capacitive-controlled process and 27.8% via a diffusion-controlled process. In addition, the capacitive/diffusion processes for the Ni-NP electrode at 5 to 60 mV/s is shown in Figure 5d. This shows that as scan rates increase, the capacitive-controlled process rises, indicating that the capacitive process dominates the overall capacitance, especially when scan rates are high.

**Figure 6:**
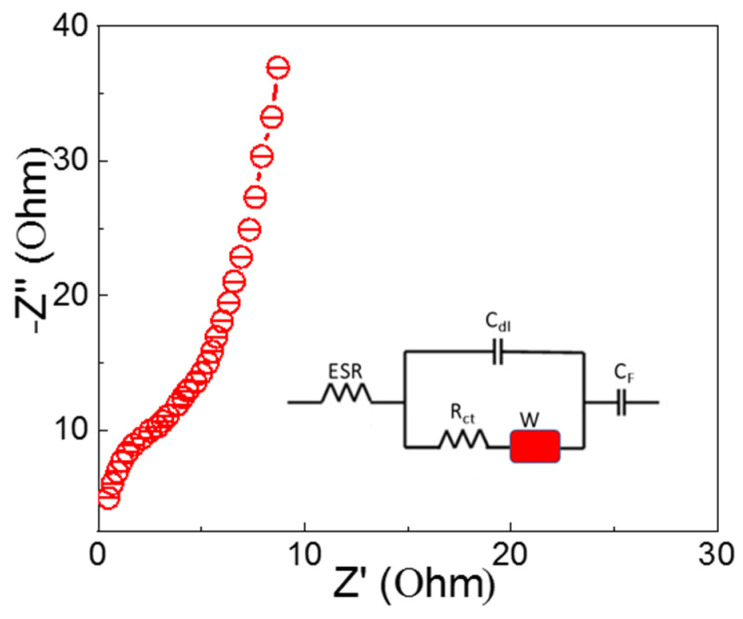
The Nyquist plot was obtained at frequencies ranging from 100 kHz all the way down to 0.01 Hz.

To gain a better understanding of the kinetic feature of the ion diffusion that is responsible for the charge storage property of the electrode, an electrochemical impedance spectrum (EIS) investigation was carried out. Figure 6 provides a visual representation of the Nyquist plot for the Ni-NP electrode. Any absence of a semicircular zone in the plot is indicative of low faradaic resistances in the electrolyte. The Nyquist plot shown in Figure 6 initially intersects the real axis at 45°. This may be due to the Warburg impedance, which occurs in a porous electrode when an electrolyte ion accesses it. A straightforward EDLC system can be identified by a vertical curve in the lower frequency zone. The Nyquist plot was well-represented by an equivalent circuit, which can be seen in the inset of Figure 6. The following equation describes this equivalent circuit [52].

(8) $$Z=R_{s}+\frac{1}{J_{w}C_{DL}+\frac{1}{R_{CT}+W_{O}}}-j\frac{1}{wC_{F}}$$

ESR is the equivalent series resistance, which comprises the resistance of the electrode materials, electrolytes, current collectors, and contact resistance; *R_ct_* is the charge transfer resistance; *C_DL_* is the double layer capacitance; *C_F_* is the faradaic capacitance; and *W_o_* is the finite-length Warburg diffusion element, which is expressed as A/(*j*)^n^, where A is the Warburg coefficient, *w* is the angular frequency, and n is an exponent [52].

**Table 1:** A comparison of Ni-NP electrode with other already published nanostructured electrode materials for SCs.

Sr. No	Electrode Material	Electrolyte	Capacitance (F/g)	Current Density (A/g)	Retention (%)	No Cycles (n)	E (Wh/kg)	P (W/kg)	Ref.
1.	Ni-NPs	KOH	730	1	92.4	5000	36.5	438.11	This work
1.	Ni-NPs	KOH	416.6	1	--	--	--	--	[53]
2.	PPy/Ni	KOH	488	0.25	65	1000	--	--	[54]
3.	NiO	KOH	116	1	84	2000	--	--	[55]
4.	MnO_2_@NGO	KOH	360	0.5	90	6000	--	--	[56]
5.	MnO_2_-CNFs	Na_2_SO_4_	324.55	0.5	62	1000	--	--	[57]
6.	Fe_3_O_4_ NPs/RGO	KOH	241	1	79.2	1000	--	--	[58]
7.	Co NP/RGO	KOH	370.7	0.5	90.4	2000	--	--	[59]

## 4. Conclusions

To conclude, we have demonstrated a unique synthetic approach called microemulsion to fabricate Ni-NPs. The assembly of the Ni-NPs ensures that the electrode is fully electrochemically used in charge storage since it promotes quick electron and ion transit. As a high-rate positive electrode for SC in 6 M KOH aqueous electrolyte, the Ni-NP electrode has several reactive sites and rapid ion diffusion channels. As a result, the Ni-NP electrode displayed good charge storage capabilities, with a capacitive method of 72.4% at 10 mV/s. Furthermore, Ni-NPs show a capacitance of 730 F/g at 1 A/g. Furthermore, even after 5000 cycles, the Ni-NP electrode retains 92.4% of its initial capacitance, showing its potential for use in the developing field of renewable energy. These findings demonstrate that Ni-NP electrodes are promising with a controllable electrochemical performance, with the potential to commercialize for the development of sophisticated SCs.

## Data Availability

Not applicable.
